# Cardiac Stem Cells for Myocardial Regeneration: They Are Not Alone

**DOI:** 10.3389/fcvm.2017.00047

**Published:** 2017-07-17

**Authors:** Yin Yee Leong, Wai Hoe Ng, Georgina M. Ellison-Hughes, Jun Jie Tan

**Affiliations:** ^1^Advanced Medical and Dental Institute, Universiti Sains Malaysia, Penang, Malaysia; ^2^Centre for Human and Aerospace Physiological Sciences, King’s College London, London, United Kingdom; ^3^Centre for Stem Cells and Regenerative Medicine, King’s College London, London, United Kingdom

**Keywords:** myocardial regeneration, cardiac stem and progenitor cells, synergy, interactions, cell therapy, cardiac tissue engineering

## Abstract

Heart failure is the number one killer worldwide with ~50% of patients dying within 5 years of prognosis. The discovery of stem cells, which are capable of repairing the damaged portion of the heart, has created a field of cardiac regenerative medicine, which explores various types of stem cells, either autologous or endogenous, in the hope of finding the “holy grail” stem cell candidate to slow down and reverse the disease progression. However, there are many challenges that need to be overcome in the search of such a cell candidate. The ideal cells have to survive the harsh infarcted environment, retain their phenotype upon administration, and engraft and be activated to initiate repair and regeneration *in vivo*. Early bench and bedside experiments mostly focused on bone marrow-derived cells; however, heart regeneration requires multiple coordinations and interactions between various cell types and the extracellular matrix to form new cardiomyocytes and vasculature. There is an observed trend that when more than one cell is coadministered and cotransplanted into infarcted animal models the degree of regeneration is enhanced, when compared to single-cell administration. This review focuses on stem cell candidates, which have also been tested in human trials, and summarizes findings that explore the interactions between various stem cells in heart regenerative therapy.

## Introduction

**Figure 1:**
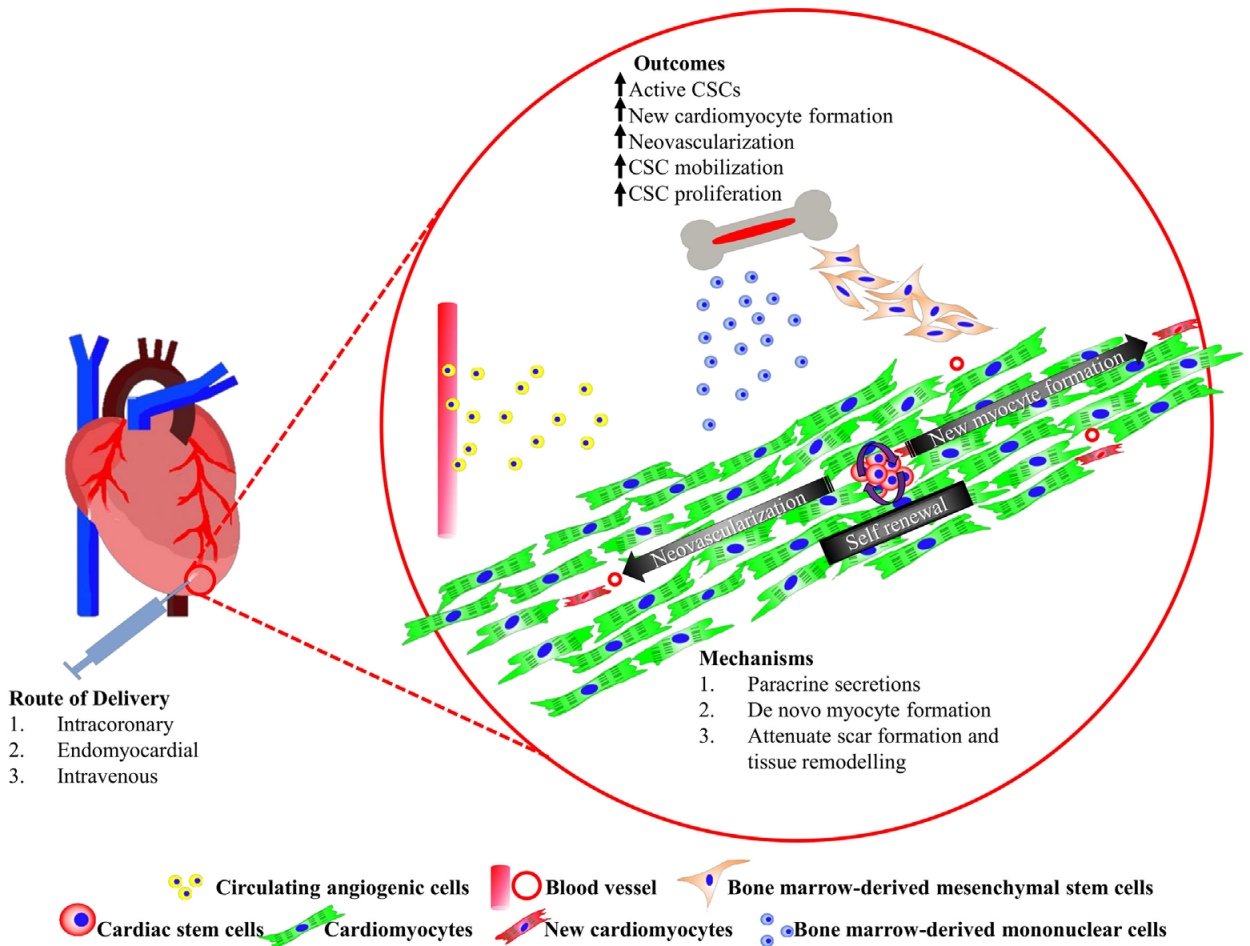
Summary of cardiac stem/progenitor cells and their interaction with bone marrow-derived cells promoting heart regeneration.

Cardiovascular disease remains the number one, non-communicable killer disease, which recorded a mortality rate of 17.5 million in 2012, and was accounted for 46.2% of all reported deaths worldwide in 2014 (1). Myocardial infarction (MI) is a common cause of heart failure (HF) due to a consequence of partial or complete occlusion of the coronary artery, which diminishes the delivery of oxygen and nutrient supply to the myocardium where the vessel serves (2). Approximately 25% of myocardial infarcted patients suffer from severe left ventricular dysfunction and are at risk of progressive heart remodeling (3). Conventional pharmacological approaches with drugs, such as thrombolytic agent, β-blocker, and angiotensin-converting enzyme inhibitor, is often the first non-invasive treatment option offered to patients. However, in more severe cases, ST-elevated myocardial infarction (STEMI), a more invasive balloon angioplasty, and stent insertion may be recommended to achieve myocardial reperfusion. Highly invasive coronary artery bypass grafting procedure is only recommended if severe, irreversible coronary occlusion is evident. These approaches had shown to alleviate the symptoms of the disease and improve the patients’ quality of life. Nevertheless, none of these therapies were able to remove the fibrotic scar or replace the lost myocardium with new functional cardiomyocytes. The presence of the akinetic tissue restricts the overall cardiac performance, forcing the remaining myocytes to increase contractility to maintain adequate cardiac output. These events trigger abrupt alterations in cardiac architecture and cause cardiomyocyte hypertrophy, further myocyte loss, thinning of the ventricular wall, weakening of contractility, and an eventual cease in function of the cardiomyocytes (4). To date, heart transplantation is the only curative option. Although there are survivors from successful heart transplantations, the long waiting time, high patient-to-donor ratio, high incidence of post-procedural complications, and limited number of transplantable hearts prompt an urgent need for an alternative solution. Stem cell-based therapies are fast becoming an attractive and highly promising treatment for heart disease and failure. The most common types of stem cell candidates, which had been tested in clinical trials thus far, are derived mainly from the bone marrow. In this review, we will discuss the basic discovery and current progress of the candidate cells in human cardiac regenerative therapy, and the potential to combine multiple cell types for regenerating complex components that make up the myocardium (Figure 1). Finally, we touch on an emerging prospective application in heart tissue engineering.

## Bone Marrow-Derived Mononuclear and Hematopoietic Stem Cells (HSCs)

The discovery of recipient-derived cardiomyocytes in sex-mismatched donor hearts after bone marrow transplants spiked the interest of using bone marrow cells for cardiac cell therapy (5–7). Bone marrow mononuclear cells (BMNCs) were the first hematopoietic cells selected for this purpose, because of their availability and feasibility to be isolated from patients through bone marrow aspiration (8). In fact, the *in vitro* procedure involved minimal manipulation for clinical transplantation, making it the most favorite cell candidate in initial cardiac repair clinical trials. Nevertheless, most clinical studies observed a marginal, yet clinically significant, improvement in cardiac function after injection with BMNCs (Table 1). Despite evidences that showed the BMNCs contribute to angiogenesis (9) and neovascularization (10) by secreting paracrine factors, their capability of cardiomyogenic differentiation *in vivo* remains skeptical. The earliest study, where lineage-negative (Lin^−^), c-kit-positive (c-kit^+^), EGFP + HSCs were injected into the contracting wall bordering the infarct in mice, showed newly formed myocardium, comprised cardiomyocytes and vasculature, occupying 68% of the infarcted portion of the ventricle 9 days after transplanting the bone marrow cells (11). These findings failed to be replicated by others. Murry et al. (12) tracked the fate of HSCs (c-kit^+^, Lin^−^) after 145 transplants into normal and injured adult mouse hearts and found no trans-differentiation of HSCs into cardiomyocytes (12). Moreover, Balsam and colleagues showed that when GFP^+^Lin^−^c-kit^+^ HSCs were injected into infarcted mouse hearts, abundant GFP^+^ cells were detected in the myocardium at 10 days, with few cells detectable at 30 days (13). It was found that the GFP^+^ cells did not express cardiac tissue-specific markers, but expressed the hematopoietic marker CD45 and myeloid marker Gr-1, representing mature hematopoietic fates.

**Table 1:** List of clinical trials using bone marrow mononuclear cells.

Study	Number of patients	Type of patients	Duration (months)	Imaging modality	Changes in left ventricular ejection fraction (LVEF)			Reference
					Placebo	Treated	% Changes in LVEF (treated)	
TOPCARE-AMI (2002)	20	Acute MI	4	LV angiography Echocardiography PET	51.0 ± 10.0–53.5 ± 7.9%	51.6 ± 9.6–60.1 ± 8.6%	+8.5*	(14)

TOPCARE-AMI (2004)^a^	59	Acute MI	4	LV angiography Echocardiography MRI	50.0 ± 10.0–58.0 ± 10.0%	49.0 ± 10.0–57.0 ± 10.0%	+8*	(15)

BOOST (2004)	60	STEMI	6	Cardiac MRI	51.3 ± 9.3–52.0 ± 12.4%	50.0 ± 10.0–56.7 ± 12.5%	+6.7*	(16)

BOOST (2006)^a^	60	STEMI	18	Cardiac MRI	51.3 ± 9.3–54.4.0 ± 13.0%	50.0 ± 10.0–55.9 ± 14.7%	+5.9	(17)

REPAIR-AMI (2006)	204	Acute MI	4	LV angiography	46.9 ± 10.4–49.9 ± 13.0%	48.3 ± 9.2–53.8 ± 10.2%	+10.5*	(18)

LEUVEN-AMI (2006)	67	STEMI	4	MRI PET Echocardiography	46.9 ± 8.2–49.1 ± 10.7	48.5 ± 7.2–51.8 ± 8.8%	+3.3	(19)

ASTAMI (2006)	97	STEMI	6	Echocardiography SPECT MRI	46.9 ± 9.6–49.0 ± 9.5%	45.7 ± 9.4–48.8 ± 10.7%	+3.1	(20)

TCT-STAMI (2006)	20	Acute MI	6	Echocardiography SPECT	58.2 ± 7.5–56.3 ± 3.5%	53.8 ± 9.2–58.6 ± 9.9%	+4.8*	(21)

TOPCARE-CHD (2007)	121	Chronic post-infarction HF	3	LV angiography	N/A	39.9 ± 11.4–41.7 ± 11.9%	+1.8*	(22)

Gowdak (2008)	10	Severe coronary artery disease	12	MRI Echocardiography	N/A	63.0 ± 14.0–67.0 ± 13.0%	+4	(23)

FINCELL (2008)	80	STEMI	6	Echocardiography LV angiography	57.0 ± 10.0–56.0 ± 10.0%	56.0 ± 10.0–60.0 ± 8.0%	+4*	(24)

HEBE (2008)	26	Acute MI	12	MRI	N/A	45.0 ± 6.3–47.2 ± 6.5%	+2.2*	(25)

BOOST (2009)^a^	60	STEMI	61	cMRI	51.3 ± 9.3–48.1 ± 12.9%	50.0 ± 10.0–47.5 ± 16.7%	−2.5	(26)

ASTAMI (2009)^a^	100	STEMI	36	Echocardiography MRI	46.9 ± 9.6–46.8 ± 8.6%	45.7 ± 9.4–47.5 ± 9.0%	+1.8	(27)

REGENT (2009)	200	STEMI	6	MRI Echocardiography LV angiography	N/A N/A	37.0–40.0% (non-selected BMC) 35.0–38.0% (CD34-CXCR4 BMC)	+3* (for both groups)	(28)

Traverse (2010)	40	STEMI	6	Echocardiography MRI	48.6 ± 8.5–57.0 ± 13.4%	49.0 ± 9.5–55.2 ± 9.8%	+6.2	(29)

BONAMI (2010)	101	Acute MI	3	RNA MRI Echocardiography SPECT	37.0 ± 6.7–41.3 ± 9.0%	35.6 ± 7.0–38.9 ± 10.3%	+3.3	(30)

REPAIR-AMI^a^ (2010)	204	Acute MI	24	LV angiography MRI	48.7–43.6%	45.4–50.1%	+4.7*	(31)

FOCUS-HF (2011)	30	Ischemic HF	6	Echocardiography SPECT LV angiography	40.0 ± 3.2–40.9 ± 8.5%	37.5 ± 8.2–42.0 ± 14.4%	+4.5*	(32)

HEBE (2011)^a^	200	Acute MI	4	MRI	42.4 ± 8.3–46.4 ± 9.2%	43.7 ± 9.0–47.5 ± 9.9%	+3.8*	(33)

Late TIME (2011)	87	Acute MI	6	Echocardiography MRI	45.3 ± 9.9–48.8 ± 7.8%	48.7 ± 12.0–49.2 ± 13.0%	+0.5	(34)

TOPCARE-AMI^a^ (2011)	55	Acute MI	60	MRI	N/A	46.0 ± 10.0–57.0 ± 10.0%	+11*	(35)

TIME (2012)	120	Acute MI	6	MRI Echocardiography	44.5 ± 10.8–47.8 ± 13.6%	45.1 ± 10.6–48.3 ± 13.3%	+3.2	(36)

Antonitsis (2012)	9	Ischemic cardiomyopathy	12	Echocardiography SPECT	N/A	31.3 ± 6.5–52.5 ± 8.9%	+21.2*	(37)

FOCUS-CCTRN (2012)	92	Chronic HF	6	SPECT	32.3–31.0%	34.7–36.1%	+1.4	(38)

SWISS AMI (2013)	200	STEMI	4	MRI	40.0 ± 9.9–38.7 ± 17.3%	36.5 ± 9.9–37.9 ± 10.3% (early injection—5–7 days post-MI) 36.3 ± 8.2–37.4 ± 9.7% (late injection—3–4 weeks post-MI)	+1.4 (early injection—5–7 days post-MI) +1.1 (late injection—3–4 weeks post-MI)	(39)

*N/A, not applicable (Placebo group was not included in trial); HF, heart failure; MI, myocardial infarction; LV, left ventricular; STEMI, ST-elevated myocardial infarction; PET, positron emission tomography; MRI, magnetic resonance imaging; SPECT, single-photon-emission computed tomography; RNA, radionuclide angiography*.

*^a^Follow-up studies*.

*Significant improvement in LVEF (*p* < 0.05).

More recently, van Berlo et al. (40) generated c-kit^cre^-IRES-eGFP knocked-in mice to revisit the fate of c-kit^+^ cells in development and following injury (40). They found that most eGFP-c-kit^+^ cells were mainly non-myocytes in the developing and injured adult heart. Indeed, c-kit^+^ cells largely adopted an endothelial lineage phenotype in the developing or infarcted heart, and rarely became cardiomyocytes (41, 42). While these models set out to tag all c-kit^+^ cells in the organism, questions were raised over the fidelity of the model and reporter gene to successfully recombine the endogenous, resident cardiac stem, and progenitor cells, which also express c-kit (43).

## Bone Marrow-Derived Mesenchymal Stem Cells

Mesenchymal stem cells, or also known as mesenchymal stromal cells (MSCs), are a subset of bone marrow-derived stem cells that have plastic adherence characteristics, express CD105, CD73, and CD90 but not CD34, CD45, CD14 or CD11b, CD79α or CD19, and HLA-DR, and possess the ability to form adipocytes, chondrocytes, and osteoblasts *in vitro* (44). As MSCs express low MHC Class I and are lacking MHC Class II (45), the phenotype confers the capability of evading host immune responses and hence enables the cells for allogeneic transplantation (45). Several *in vivo* studies showed improvements in myocardial function despite low rates of MSC engraftment and differentiation (46, 47). Although trans-differentiation of MSCs into cardiomyocytes was achievable by using demethylating chemicals (48, 49) or by coculturing with rodent myocytes *in vitro* (50, 51), the event *in vivo* had been reportedly low (52). Furthermore, electrophysiological analysis revealed that differentiated myocytes did not possess similar electrical properties to a functional cardiomyocyte (53). Hence, the main regenerative function of MSCs was largely confined to its secretome, which contained a plethora of factors with cardioprotective effects, or stimulants that activate endogenous repair mechanisms including the resident cardiac stem and progenitor cells (54, 55).

Many trials had been conducted to examine the therapeutic efficacy of MSCs in regenerating damaged human hearts at different severities, either with autologous or allogeneic cell sources (Table 2). In POSEIDON, transendocardial-administered allogeneic BM-MSCs attenuated the progressive heart remodeling, reduced the scar mass, and improved the early enhancement defect and sphericity index in ischemic cardiomyopathic patients, and the effects were greater with a lower cell dose (20 million), as compared to a higher dose (200 million) (56). The injected allogeneic MSCs did not trigger immune responses in recipients, and the observed benefits were mostly similar to autologous MSCs (56). However, both allogenic and autologous MSC-treated groups did not show significant improvements in ejection fraction. In contrast, the phase 2, placebo-controlled randomized MSC-HF trial reported encouraging results, which demonstrated that HF patients who received a high number of intramyocardially delivered autologous MSCs showed greater functional improvements in the ischemic heart after 12 months (57). They also suggested a possible correlation between cell dose and disease severity. Through a longer, 2-year follow-up, the phase 1 pilot study MESAMI revealed similar benefits from intramyocardial MSC injection in patients with chronic ischemic cardiomyopathy, albeit with a smaller sample size of 10 (58).

**Table 2:** Clinical trials using bone marrow-derived mesenchymal stem cells.

Study	Number of patients	Type of patients	Duration (months)	Imaging modality	Changes in left ventricular ejection fraction (LVEF)			Reference
					Placebo	Treated	% Changes in LVEF (treated)	
Chen (2004)	69	Acute MI	6	Echocardiography PET	48.0 ± 10.0–54.0 ± 5.0%	49.0 ± 9.0–67.0 ± 3.0%	+18*	(59)

Hare (2009)	53	Acute MI	6	Echocardiography MRI	48.7–56.1%	50.4–56.9%	+6.5	(60)

POSEIDON (2012)	30	Ischemic cardiomyopathy	13	Echocardiography	N/A	27.85–29.5% (allogeneic) 26.23–28.53% (autologous)	+1.65 (allogeneic) +2.3 (autologous)	(56)

PROMETHEUS (2014)	6	Ischemic left ventricular dysfunction secondary to MI	18	MRI	N/A	41.2 ± 4.9–51.3 ± 5.4.0%	+10.1*	(61)

SEED-MSC (2014)	80	Acute MI	6	Echocardiography SPECT	49 ± 11.7–55 ± 11.8%	52.3 ± 9.3–53.9 ± 10.2%	+1.6*	(62)

TAC-HFT (2014)	65	Ischemic cardiomyopathy	12	MRI CT Echocardiography	N/A	28.1 ± 0.8–35.7 ± 9.0%	+7.6	(63)

MSC-HF (2015)	55	Ischemic HF	6	Echocardiography MRI CT	25.1–23.8%	28.2–33.2%	+5*	(57)

MESAMI (2016)	10	Ischemic cardiomyopathy	12	Echocardiography SPECT	N/A	29.4 ± 2.0–35.7 ± 2.5%	+6.3*	(58)

*N/A, not applicable (Placebo group was not included in trial); HF, heart failure; MI, myocardial Infarction; LV, left ventricular; PET, positron emission tomography; MRI, magnetic resonance imaging; SPECT, single-photon-emission computed tomography; CT, cardiac tomography; MSC, mesenchymal stromal cell*.

*Significant improvement in LVEF (*p* < 0.05).

## Endogenously Derived Resident Cardiac Stem and Progenitor Cells

### c-kit^+^ Cardiac Stem Cells (CSCs)

The first reported primitive CSCs present in the heart were identified and isolated based on the expression of stem cell factor receptor CD117 or c-kit. c-kit^+^ CSCs are also positive for Sca-1 (60 ± 10% of c-kit^+^ eCSCs are also Sca-1^+^), MDR-1 (ABCG2), and other markers identified on adult cardiac stem and progenitor cell populations, such as CD105, CD166, PDGFrα, and CD90. c-kit^+^ CSCs do not express CD34, CD31, CD45, or tryptase, distinguishing them from c-kit^+^ endothelial (progenitor) cells and mast cells (64, 65). CSCs are multipotent, self-renewing, and capable of forming cardiomyocytes, smooth muscle cells, and endothelial cells (64, 65), and their turnover was coupled with cellular homeostasis in the heart (66). In the adult heart, most of the CSCs were found to reside in the atrium and the ventricular apex, albeit at a very low density (1 cell per every 10,000 myocytes) (64). Owing to the scarcity of the CSCs, an optimized protocol had been developed to isolate and characterize these cells (67). CSCs can be propagated over long-term culture and maintained in an undifferentiated, self-renewing, stable state, without showing evidence of senescent growth arrest or abnormal karyotype (68). Preclinical studies showed that these c-kit^+^ CSCs regenerated both the hearts of rats (64, 69) and mice (65, 70) post-infarction *via* the formation of new myocytes and vasculature, and protected the preexisting cardiomyocytes from apoptosis through the secretion of IGF-1 (71, 72). The significance of CSCs was further highlighted in an elegant experiment which employed an animal model by which the proliferating cells in the damaged heart were totally ablated using 5-flurouracil, which lead to a blunted the recovery of the injured heart (69). However, the recovery was reversed, both anatomically and functionally, through the administration of c-kit^+^ clonogenic CSCs, suggesting their indispensable role in restoring and initiating myocardial repair and regeneration in response to injury.

c-kit^+^ CSCs have been tested in human trials (Table 3). The phase 1 stem cell infusion in patients with ischemic cardiomyopathy (SCIPIO) trial showed that intracoronary administration of c-kit^+^ CSCs (1 million) increased the left ventricular ejection fraction (LVEF) by 7.6 and 13.7% with decreased infarct size of 6.9 and 7.8 g after 4 and 12 months, respectively (73, 74). A study was also performed to address the safety of intracoronary infusion of 20 million c-kit^+^ CSCs into swine hearts (75). The results showed neither renal and liver damage nor further myocardial injury due to microembolism. Nonetheless, the cell retention in the myocardium remained low despite the high number of infused cells.

**Table 3:** Clinical trials using cardiac stem cells.

Study	Number of patients	Type of patients	Duration (months)	Imaging modality	Changes in left ventricular ejection fraction (LVEF)			Reference
					Placebo	Treated	% Changes in LVEF (treated)	
SCIPIO (2011)	23	HF	4	Echocardiography MRI	30.1 ± 2.4–30.2 ± 2.5%	30.3 ± 1.9–38.5 ± 2.8%	+8.2*	(73)
SCIPIO^a^ (2012)	33	HF	4 and 12	Echocardiography MRI	N/A	27.5 ± 1.6–35.1 ± 2.4% (4th month) and 41.2 ± 4.5% (12th month)	+7.6* (4th month) + 13.7 (12th month)	(74)
CADUCEUS (2012)	25	MI	6	MRI	39–44.8%	38–43.4%	+5.4	(76)
CADUCEUS^a^ (2014)	25	MI	12	MRI	42.5 ± 11.1–48.2 ± 11.4%	42.4 ± 8.9–48.2 ± 10.3%	+5.4	(77)

*N/A, not applicable (Placebo group was not included in trial); HF, heart failure; MI, myocardial infarction; LV, left ventricular; MRI, magnetic resonance imaging*.

*^a^Follow-up studies*.

*Significant improvement in LVEF (*p* < 0.05).

### Cardiospheres and Cardiosphere-Derived Cells (CDCs)

Cardiospheres are 20–150 µm cellular spheres, which are generated from the explant outgrowth cells of heart biopsies (65, 78). These cardiospheres supposedly consist of CSCs that reside in the core and cardiac lineage committed cells (e.g., myofibroblasts) and differentiated cells (vascular smooth muscle cells, endothelial cells), which comprise the outer layer of the spheres (65). The three-dimensional microenvironment of cardiospheres had been shown to protect the CSCs from oxidative stress as well as maintain their stemness and function (79). When these cardiospheres were expanded on fibronectin, the CDCs became highly proliferative in the monolayer and were clonogenic and multipotent, *in vitro* (80). This enables fast and efficient expansion of the CDCs for heart therapy, with retained regenerative potential (78, 81, 82). The therapeutic effects of CDCs had also been demonstrated in *in vivo* studies, ranging from small-to-large animal models (81, 83, 84) and in human trials (76, 85). CDCs showed potential in reducing infarct size, improving LVEF and cardiac hemodynamics in infarcted animal models (81, 83), which could be maintained for up to 16 weeks (82). The positive observation in *in vivo* studies led to the initiation of a randomized phase 1 clinical trial, known as the cardiosphere-derived autologous stem cells to reverse ventricular dysfunction study or the CADUCEUS trial (76). The trial showed significant reductions in scar mass (8.4 g in the first 6 months and 12.9 g after a year) but no differences in the LVEF.

## Relationship Between Bone Marrow-Derived Cells and Resident CSCs

Cardiospheres and CDCs represent a mixed cell population, which employs an assortment of heterogeneous cells and this heterogeneity sparks the idea of employing synergistic effects between various cells to aid CSCs to perform better for cardiac regeneration.

Mononuclear bone marrow cells had been shown to benefit the injured myocardium after their administration, but the effect was then concluded as not sustainable. Paracrine signaling is a generally accepted explanation for the mechanism of repair, regeneration, and modest improvement in cardiac function. Loffredo et al. (86) conducted a sophisticated experiment using bitransgenic MerCreMer ZEG mice to study the degree of new myocyte formation after induced injury in the heart, following BMDC transplantation (86). In this model, all cardiomyocytes were permanently shifted to express GFP from β-galatosidase (β-gal) by a pulse treatment of 4-OH tamoxifen, and all new myocytes were identified as non-GFP expressing β-gal-positive cells. The study revealed that the number of new myocytes was greater in subjects treated with c-kit^+^ bone marrow MNCs. This coincided with increased resident GATA4^+^Nkx2.5^+^ cardiac progenitors, which was not observed when the subjects were given bone marrow MSCs. Moreover, Hatzistergos et al. (54) used GFP-transduced MSCs and when transendocardially injected them into the infarcted heart of the Yorkshire swine and showed increased GFP^−^ c-kit cells by 2- and 15-fold in the infarcted and border regions, respectively (54). These cells coexpressed MDR-1 and GATA4, suggesting that they were of endogenous CSC origin. These findings were consistent with the *in vitro* data which showed that greater c-kit^+^ CSCs were mobilized from heart explant cultures in the presence of MSC feeder layers (87). In addition to the activation of the endogenous pool of CSCs, MSCs prompted cardiomyocyte proliferation, which correlated with an increased number of cardiomyocytes expressing serine 10 phosphorylated histone H3, a mitotic marker indicative of cell cycling (54). The regenerative capability of MSCs was further confirmed with a study by Suzuki et al. (88), which discovered the ability of MSCs in mobilizing CD133 and c-kit^+^ bone marrow cells as well as stimulating myocyte proliferation in chronic hibernating myocardium (88). Indeed, the administration of MSCs was found to drive the increment of c-kit^+^/CD133^−^, c-kit^+^/CD133^+^ progenitor cells, and Ki67^+^ and phospho-histone H3^+^ cardiomyocytes.

The synergistic effects between MSCs and heart-derived, resident c-kit^+^ CSCs were further confirmed in two studies where cotransplantation of both cell types showed greater amelioration in improving cardiac performance and scar size post-infarction (89, 90). Both transepicardial and transendocardial administrations of either xenogenic or autologous MSCs showed greater scar reductions and global heart function restorations as compared to single-cell administration in swine model, which illustrates the interaction between MSCs and CSCs in enhancing the regeneration of the heart post-infarction.

## CSCs Relationship with Other Cells

### Telocytes

Telocytes, which were first described in 2009 as interstitial Cajal-like cells, are peculiar stromal cells that were recently found to reside in the interstitium in all heart layers (91–93). These cells express vimentin and CD34, with several reports that showed coexpressions with c-kit or PDGFR-β markers (94). Of note, the unique phenotype that distinguishes telocytes from other interstitial cells is the distinct and very fine cellular prolongation called telopodes. The average length of these telopodes could extend from a few ten to hundred microns. Transmission electron microscopic analysis showed that most of the telocytes intermingled with adjacent cardiomyocytes and precursors of telopodes forming an organized myocyte cluster that was integrated in the myocardium (95). Furthermore, changes in number of telocytes have also found to be associated with severe alterations in heart matric architecture (96), and transplantation of telocytes into injured rat hearts had also shown improved functions (97). Although there is no direct evidence that demonstrates how telocytes functionally influence CSC activity *in vivo*, the distribution and organization of these telocytes in the myocardial interstitium, however, support the notion that they may be an important “nurse” cell in the CSC niche that governs endogenous precursors and immature cardiac myocytes in heart development and regeneration (98).

### Epicardial-Derived Cells (EPDCs)

The epicardium consists of a unique population of cells that originated from the proepicardial organ expressing WT1, Tbx18, and retinaldehyde dehydrogenase2. These cells enveloped the developing heart and formed distinct layers of epicardium and subepicardial mesenchyme, which promoted cardiomyocyte proliferation, triggered myocardial expansion to generate thick myocardium during heart development (99, 100). EPDCs contributed to the majority of non-myocyte support cells, such as cardiac fibroblasts and smooth muscle cells and their invasion to the myocardium and endocardium was accomplished *via* the epithelial–mesenchymal transition (101). A study conducted by Winter et al. (102) showed that EPDCs facilitated cardiomyocyte progenitor cell (CMPC) proliferation under hypoxic conditions (1% O_2_) in coculture, albeit with decreased cell motility (102). Coculture of both EPDCs and CMPCs produced increased angiogenic factors, such as VEGF and PDGF-BB. *In vivo*, an MRI study showed an improvement in ejection fraction and a significant decrease in end systolic and diastolic volumes when both cells were administered. Significantly higher endothelial densities at the border and infarcted zones were also observed, with preserved ventricular wall thickness. However, *in vivo* results showed that there were little to no cell engraftment or differentiation in the infarcted heart after EPDC/CMPC administration. This suggests that a paracrine interaction may be the main reason for the improved heart function, and the CMPCs were enhanced through the secretion of growth factors by EPDCs (102).

### Circulatory Angiogenic Cells (CACs)

Surviving hostile environments primarily requires the establishment of perfusion and revascularization of the infarct regions. Hence, the vascular network within the injected region is key to cell survival. CACs, or early outgrowth endothelial progenitor cells, were considered blood-derived cells that play a role in both vasculogenesis and angiogenesis in promoting myocardial repair, mainly through paracrine interaction (103). A study by Latham et al. (103) demonstrated that conditioned medium from CAC–CSC cocultures showed greater capacity in mobilizing CACs and inducing tubule formation in HUVECs *in vitro*, which was attributed to the upregulation of angiogenic factors, such as angiogenin, SDF-1α, and VEGF. Echocardiography showed significant restoration of the LVEF and reduced scar formation in infarcted hearts of NOD/severe combined immunodeficient (SCID) mice following coadministration of CACs and CSCs (103). These improvements were also coupled with successful but modest smooth muscle cell, endothelial cell, and cardiomyocyte differentiation.

### Saphenous Vein-Derived Pericytes (SVPs)

Pericytes (also known as Rouget cells, mural cells, or perivascular mesenchymal precursor cells) are mesodermal cells that surround the endothelial lining in the microvasculature. These cells were highly proliferative and express neural/glial antigen 2, Sox-2, PDGFrβ, CD34, and several mesenchymal markers such as CD105, CD90, and CD44. Various studies have suggested that the transplantation of SVPs into ischemic limb was previously found to restore the regional circulatory network *via* new vessel formation in immunodeficient mice (104). Moreover, fibrotic scar, cardiomyocyte death, and vascular permeability were found to be reduced in infarcted mice myocardium that was treated with SVP, *via* microRNA-132-mediated angiogenesis (105). The relationship of the SVP with the endogenous CSC was first described by Avolio et al. (106). However, unlike the bone marrow-derived MSCs, the *in vivo* study suggested no additional benefits in restoring the ventricular function and hemodynamics when CSCs were intramyocardially cotransplanted with SVP into the infarcted heart of SCID/Beige-immunodeficient mice. Although mice that received both cells showed greater reductions in scar size, the differences were not statistically significant when compared to treatment with CSCs or SVP cells only (106).

## Cardiac Cell Therapy in Clinical Trials

**Figure 2:**
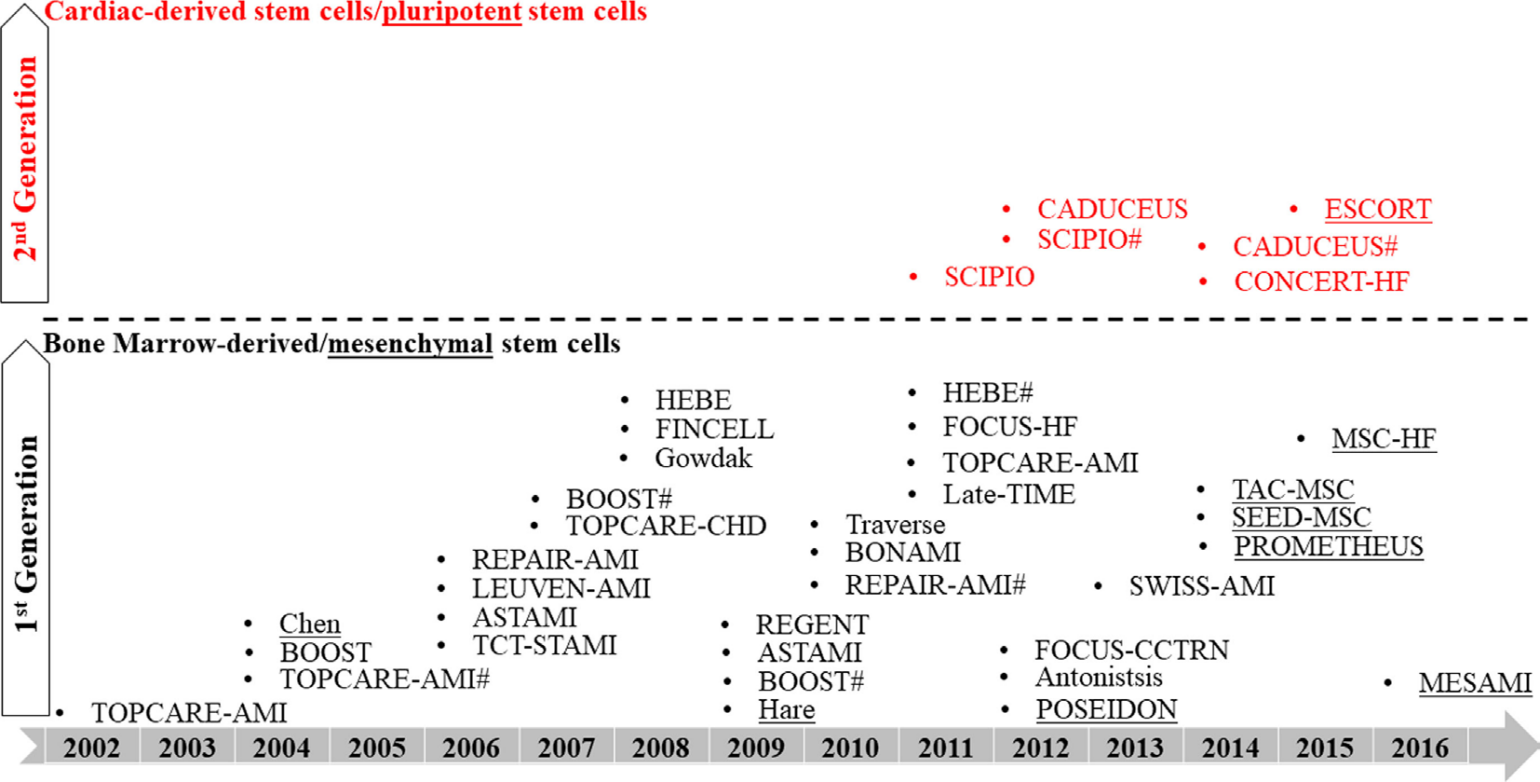
Roadmap of clinical trials using first- and second-generation cells. ^#^ indicates follow-up study.

Bone marrow-derived stem cells remain the most common, first-generation cell candidate used in clinical transplantation. A striking report by Nowbar et al. (107), who conducted a weighted regression and meta-analysis to study 49 trial reports using autologous bone marrow stem cells and outlined the discrepancies between these trials, concluded that only 10% of the human studies were performed without errors with none showing benefits from BMNCs (107). In contrast, Fisher et al. (108) performed a systemic review that excluded all the non-randomized trials, of which contributed to the majority of discrepancies outlined in Nowbar’s report, and suggested that autologous bone marrow stem cell treatment can improve HF patients’ quality of life and exercise capacity (108). Their findings are in line with a recent meta-analysis which included 48 randomized-controlled trials by Afzal et al. (109) and also agreed that bone marrow-derived cells (both BMNCs and MSCs) improved heart function in ischemic heart disease patients (109). Nonetheless, it is widely accepted that the therapeutic benefits from bone marrow-derived cells are mainly attributed to a paracrine mechanism that activates endogenous healing. Reconstituting injured myocardium with cardiomyocytes may require second-generation cardiogenic cells, the more defined, homogeneous cardiac-derived stem/progenitor cells or pluripotent stem cells, some of which have been used for clinical trials (73, 74, 76, 77, 110) (Figure 2). Careful selection of cell candidates, mode of delivery, employment of cell engraftment and enhancement strategies, in-depth investigation of mechanisms of efficacy, and clinically meaningful endpoints in future experimental studies can help to advance cardiac cell therapy (111).

## Future Directions: Emerging, State-of-the-Art Applications in Heart Tissue Engineering

Although several stem cells have been proposed to regenerate the heart, there is no consensus on the best cell type to be used in cellular therapy and the search for establishing a gold standard is still ongoing. Given the complexity of the heart, and the emptiness of the infarcted area, the regeneration process will require multiple coordinations from different therapeutic cells with synergistic functions, together with an established extracellular matrix scaffold. Some *in vivo* studies have investigated these approaches, but it has not been widely explored. It is important to realize that most experiments are conducted in two-dimensional culture systems and little is known about the survival and performance of these interactions in the three-dimensional structure. These questions lead back to the fundamental investigation of determining the optimal cell types for the engineering of tissue constructs, and their functional behaviors in three-dimensional cultures. Ott et al. (112) demonstrated a new concept of producing bio-engineered hearts by using the natural hearts from rats (112) by decellularizing the heart scaffold using detergents, then re-cellularizing through introducing neonatal cardiac cells and endothelial cells (112).

With the invention of induced-pluripotent stem (IPS) cells, the mass generation of human cardiomyocytes is no longer difficult. The challenging aspect, however, is reintroducing the cells into the construct, finding the means of ensuring their long-term survival and identifying the factors that drive their maturation. Lei Yang’s laboratory generated cardiovascular progenitors from IPS cells and attempted to reintroduce these cells into the decellularized mouse heart scaffold. The group demonstrated *ex vivo* proliferation, migration, and differentiation in the three-dimensional construct, but failed to regrow the myocardium to acquire sufficient strength for pumping fluid like the native heart (113). Another similar study by Guyette et al. (114) repopulated decellularized human hearts with cardiomyocytes derived from IPS cells. This study showed that the cardiomyocytes successfully engrafted onto the cardiac scaffolds and showed electrical conductivity and thus set the ground for the translational value of using acellular human heart matrix for complete myocardial regeneration in the future (114). A complex three-dimensional construct is an extremely promising approach for heart regeneration. However, the research is still in its infancy, and more studies are required before this technique can be translated into clinical applications.

## Author Contributions

JT and GE-H contributed to the conception and design of the review. YL, WN, and JT prepared, drafted, and wrote the manuscript. JT and GE-H wrote, critically revised, proofread, and approved the manuscript.

## Conflict of Interest Statement

JT received a research grant from CryoCord Sdn Bhd. All funders have no role in manuscript writing and funding this project. The authors declare that the research was conducted in the absence of any commercial or financial relationships that could be construed as a potential conflict of interest.
